# On the Treatment of Scarlatina

**Published:** 1811-05

**Authors:** W. Hamilton

**Affiliations:** Ipswich


*To the Editors of the Medical and Physical Journal.*

Gentlemen,

Having favoured my former communications with a place in your useful Journal, I have only to request your insertion of the following.

Feeling

Feeling extremely obliged to SENEX for his information on Scarlatina, as well as his candid remarks on my paper, I should be wanting in gratitude did I not immediately comply with his request.

"It would greatly assist us in estimating the value of Mr. Hamilton's paper on Scarlatina, to be informed whether he has met with any fatal instances from the immediate effects of the disease; or whether the cases he has given us should be regarded as comprising a summary of his whole experience on that head, and as warranting a conclusion, that his practice has been attended with universal success?"

To this I answer, that during the prevalence of the disease here (about a year and half) no fatal instance occurred under my immediate care, in which the practice alluded to was early and rigidly pursued. While I announce this success, I ingenuously confess that in two or three instances the disease terminated fatally, in all of which, being either prevented by prejudice, or called too late, the above treatment was not adopted.

No fatal instance even of the remote consequences, enumerated by your correspondent SENEX, occurred to me, where the antiphlogistic regimen had been strictly followed.

I am also much obliged to T. F. R. for his observation on the same subject, and request he will give venesection a fair trial the first opportunity, when I have little doubt of his complete conviction of its success.

I speak thus warmly in favour of phlebotomy, having met with no instance in which its effects seemed the least injurious, but on the contrary, highly beneficial even at an advanced period of the disease.

I remain, Gentlemen,
With great respect,
Your's, &c.
W. HAMILTON.

*Ipswich, April* 2, 1811.

